# Type I and III interferon responses in SARS-CoV-2 infection

**DOI:** 10.1038/s12276-021-00592-0

**Published:** 2021-05-06

**Authors:** You-Me Kim, Eui-Cheol Shin

**Affiliations:** 1 Graduate School of Medical Science and Engineering, Korea Advanced Institute of Science and Technology (KAIST), Daejeon, 34141 Republic of Korea; 2 Center for Epidemic Preparedness, KAIST, Daejeon, 34141 Republic of Korea

**Keywords:** Infectious diseases, Infection

## Abstract

Coronavirus disease 2019 (COVID-19), the current pandemic disease, is caused by severe acute respiratory syndrome coronavirus 2 (SARS-CoV-2) infection. Type I and III interferons (IFNs) are innate cytokines that are important in the first-line defense against viruses. Similar to many other viruses, SARS-CoV-2 has evolved mechanisms for evading the antiviral effects of type I and III IFNs at multiple levels, including the induction of IFN expression and cellular responses to IFNs. In this review, we describe the innate sensing mechanisms of SARS-CoV-2 and the mechanisms used by SARS-CoV-2 to evade type I and III IFN responses. We also discuss contradictory reports regarding impaired and robust type I IFN responses in patients with severe COVID-19. Finally, we discuss how delayed but exaggerated type I IFN responses can exacerbate inflammation and contribute to the severe progression of COVID-19.

## SARS-CoV-2: exploring virus-triggered immune system dysfunction

Extensive studies into how SARS-CoV-2 manipulates the immune system and influences the activity of host proteins are needed to improve treatments for COVID-19. SARS-CoV-2 evades or blocks elements of the immune system, including the antiviral activity of type I and type III interferons (IFN). You-Me Kim and Eui-Cheol Shin at the Korea Advanced Institute of Science and Technology, Daejeon, South Korea, reviewed understanding of how SARS-CoV-2 inhibits IFN responses. In infected cells, SARS-CoV-2 proteins use diverse methods to inhibit host IFN pathways, but type I IFN responses are still triggered in non-infected immune cells. The researchers believe this may explain the delayed but exaggerated type I IFN responses that contribute to the hyper-inflammation seen in critically ill patients. They call for further investigations into IFN and inflammatory responses in SARS-CoV-2 infection.

## Introduction

Severe acute respiratory syndrome coronavirus 2 (SARS-CoV-2), which causes coronavirus disease 2019 (COVID-19), is spreading globally^1,2^. The World Health Organization has declared COVID-19 a pandemic, and as of December 20, 2020, more than 76 million confirmed cases and more than 1.6 million deaths have been reported worldwide. SARS-CoV-2 infection results in a broad spectrum of clinical manifestations, from asymptomatic or mild disease to severe disease^3,4^. Although the mortality rate of COVID-19 is lower than that of severe acute respiratory syndrome (SARS) caused by SARS-CoV and Middle East respiratory syndrome (MERS) caused by MERS-CoV, it is much higher than that of influenza^5,6^. A better understanding of the immune responses against SARS-CoV-2 is needed to develop effective therapeutic strategies.

Interferons (IFNs) are potent multifunctional cytokines secreted by various cell types. In particular, type I and III IFNs play crucial roles in innate immune responses during viral infection. However, many viruses, including SARS-CoV-2, are known to inhibit type I and III IFN responses at various points, from cytokine production to receptor signaling. In addition, dysregulated IFN responses are associated with the immunopathogenesis of viral infection^5^.

In the current review, we describe the innate sensing mechanisms of SARS-CoV-2 and the mechanisms of SARS-CoV-2-mediated inhibition of IFN responses. We also discuss the dysregulation of IFN responses in the context of hyperinflammation in patients with COVID-19. Finally, we propose a hypothesis for how delayed but exaggerated type I IFN responses can contribute to the severe progression of COVID-19.

## IFNs and signaling

Originally identified as secretory factors that inhibit viral infections, IFNs are classified into three groups: types I, II, and III. Type I IFNs consist of multiple subtypes of IFN-α and a single type of IFN-β, in addition to the less well-characterized IFN-δ, -ε, -κ, -τ, -ω, and -ζ. In contrast, the type II IFN group has only a single member, IFN-γ, which is secreted by natural killer and T cells but not directly by virus-infected cells, and is therefore not described further in this review. Type III IFNs are structurally related to IL-10 family cytokines and consist of IFN-λ1 (IL-29), -λ2 (IL-28A), -λ3 (IL-28B), and -λ4^7–9^. IFN-β and IFN-λs can be secreted by any type of cell upon viral infection, whereas IFN-αs are generally produced by immune cells, particularly monocytes and dendritic cells (DCs).

**Fig. 1:**
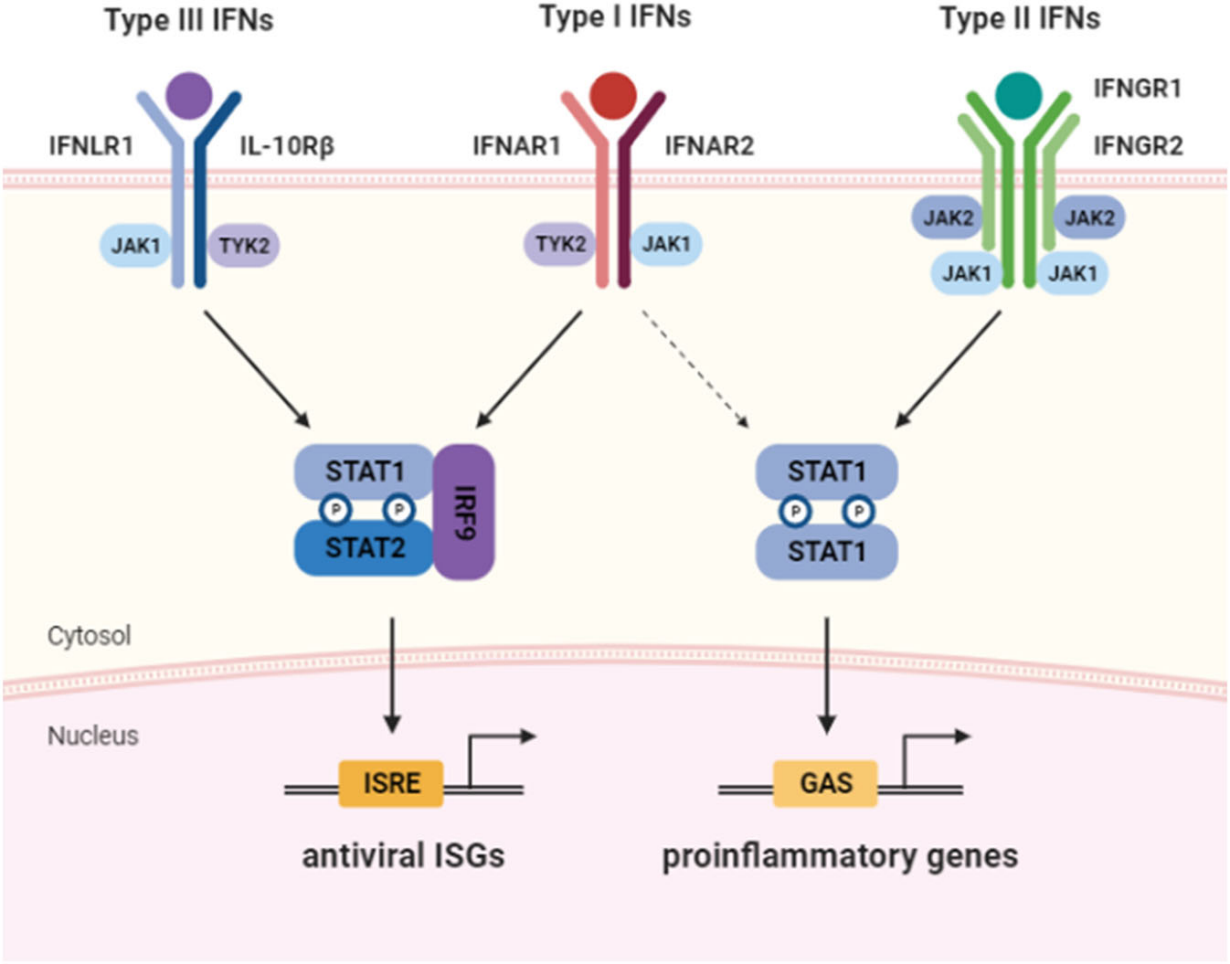
The receptors and downstream signaling pathways of type I, type II, and type III interferons (IFNs). Type I and type III IFNs bind to the heterodimeric receptor complexes IFNAR1/IFNAR2 and IFNLR1/IL-10Rβ, respectively. Upon IFN binding, the receptor-associated kinases JAK1 and TYK2 phosphorylate STAT1 and STAT2. Together with IRF9, phosphorylated STAT1 and STAT2 form a trimeric complex called IFN-stimulated gene factor 3 (ISGF3). ISGF3 subsequently enters the nucleus and binds IFN-stimulated response elements (ISREs) to promote the transcription of hundreds of IFN-stimulated genes (ISGs). Type II IFN binds to the receptor complex composed of IFNGR1 and IFNGR2 and promotes the phosphorylation of STAT1 via JAK1 and JAK2. Phosphorylated STAT1 forms homodimers, which bind gamma-activated sequences (GASs) in the nucleus and induce proinflammatory gene expression. Unlike type III IFNs, type I IFNs can also signal via STAT1 homodimers and promote proinflammatory gene expression.

Type I IFNs bind the heterodimeric receptor complex of IFNAR1 and IFNAR2 and activate the receptor-associated tyrosine kinases TYK2 and JAK1, which in turn phosphorylate STAT1 and STAT2^7–9^. Together with IRF9, phosphorylated STAT1 and STAT2 form a trimeric complex called IFN-stimulated gene factor 3 (ISGF3) that subsequently enters the nucleus to bind IFN-stimulated response elements (ISREs) and promote the transcription of hundreds of IFN-stimulated genes (ISGs; Fig. 1). A subgroup of ISGs can also be upregulated by unphosphorylated ISGF3, which is formed by high levels of unphosphorylated STAT1, unphosphorylated STAT2, and IRF9^10,11^. Many ISGs directly repress viral replication via various mechanisms, including the inhibition of viral transcription/translation and degradation of viral nucleic acids. Type I IFN receptor activation can also promote the homodimerization of STAT1, which binds gamma-activated sequences (GASs) and induces proinflammatory gene expression^7^. The type II IFN receptor, which is composed of IFNGR1 and IFNGR2, signals via JAK1 and JAK2, and IFN-γ-bound IFNGR1/2 promotes the phosphorylation and homodimerization of STAT1, resulting in the expression of GAS-regulated genes.

The type III IFN receptor is formed by IFNLR1 (also called IL-28Rα) and IL-10R2. IFNLR1 specifically binds IFN-λs, whereas IL-10R2 binding is shared by other cytokines in the IL-10 family. Similar to the type I IFN receptor, the type III IFN receptor signals via JAK1 and Tyk2 and induces ISG expression via ISGF3. Therefore, despite engaging unique receptors, type I and type III IFNs activate overlapping intracellular signaling pathways and mediate the induction of similar sets of ISGs. However, the viral infection-induced expression kinetics of type I and type III IFNs vary in vivo, and type I IFNs are usually produced earlier than type III IFNs. Moreover, type III IFN receptor expression is generally limited to epithelial cells and a subset of myeloid lineage leukocytes, whereas type I IFN receptors are ubiquitously expressed^8^. The differential receptor expression patterns and qualitative and quantitative variations in receptor signaling ultimately result in distinctive biological responses to type I and III IFNs. In general, type I IFNs trigger faster and stronger ISG induction than type III IFNs and promote the additional expression of proinflammatory cytokines and chemokines, which may cause immunopathology in situations in which the excessive responses of type I IFNs are unrestrained^8,12^.

## Innate sensing of coronaviruses

**Fig. 2:**
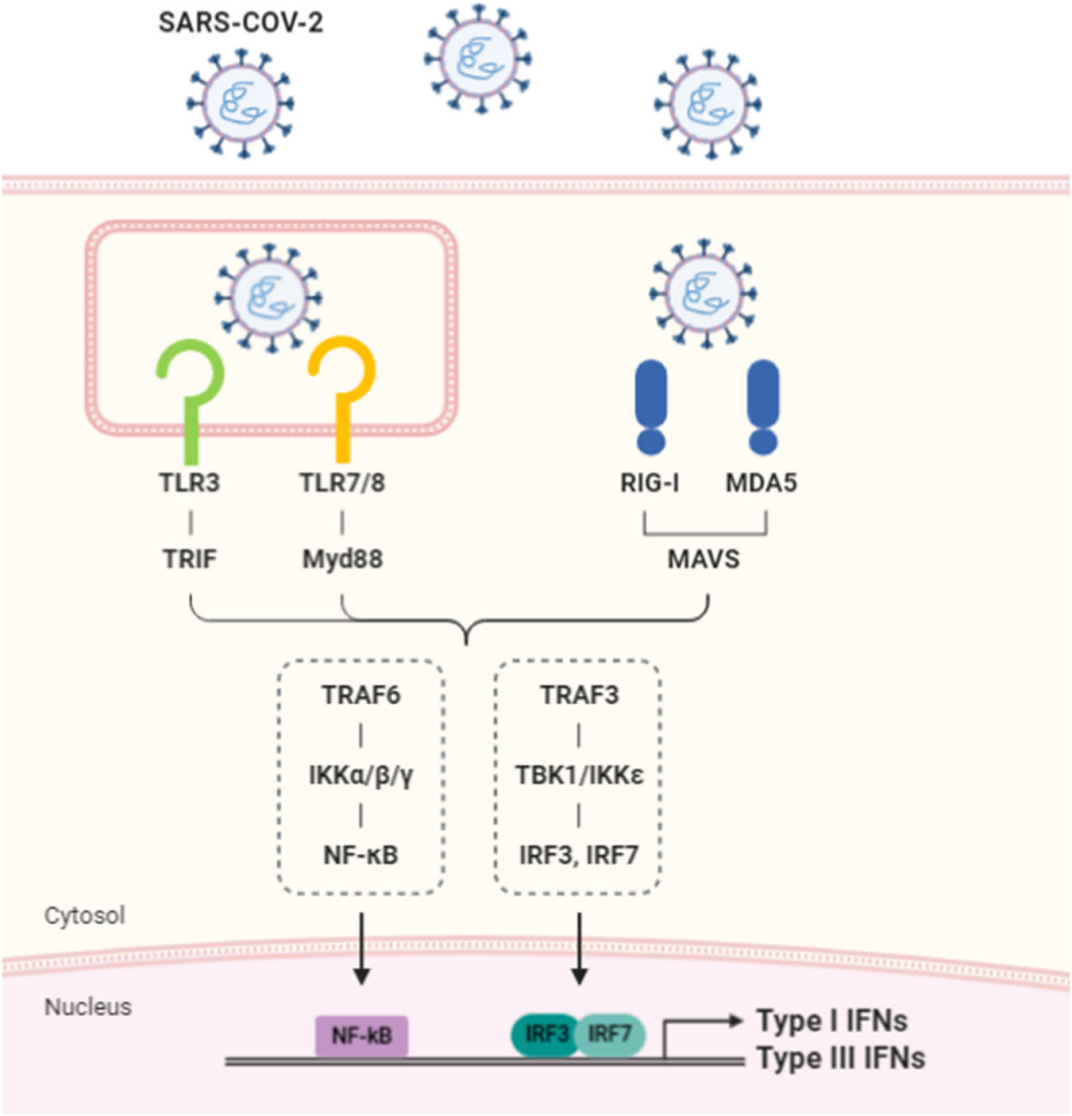
The sensing of SARS-CoV-2 by innate immune receptors and signaling pathways leading to the production of type I and type III interferons. The viral RNA genome and replication intermediates of SARS-CoV-2 can be sensed by Toll-like receptors (TLR3 and TLR7) and cytosolic RNA sensors (RIG-I and MDA-5). RNA-bound receptors induce the activation of the transcription factors NFκB and IRF3/IRF7 via the TRAF6/IKKα/β/γ and TRAF3-TBK1/IKKε pathways, respectively, and promote the expression of type I and type III interferons (IFNs).

Coronaviruses have a positive-sense single-stranded RNA (ssRNA) genome of ~30 kb^13,14^. Inside infected host cells, the viral genome is replicated by a virus-encoded RNA-dependent RNA polymerase and forms double-stranded RNA (dsRNA) replication intermediates. The ssRNA genome and dsRNA replication intermediates can be sensed by Toll-like receptors (TLRs) and RIG-I-like receptors (RLRs) in host cells (Fig. 2). RLRs, such as RIG-I and MDA-5, are expressed in most cell types and become activated by detecting viral RNAs in the cytoplasm. RIG-I recognizes viral genomic RNAs with 5′-triphosphate or short dsRNA sequences, whereas MDA-5 typically detects dsRNA structures with longer lengths^15^. Activated RIG-I and MDA-5 recruit TRAF3 via the adapter molecule MAVS (also called IPS-1, VISA, or CARDIF) and activate the kinases TBK1 and IKKε, ultimately resulting in the phosphorylation of IRF3 and transcriptional induction of type I and type III IFNs. Interestingly, it was shown that the intracellular location of the MAVS protein critically determines which IFNs are produced^16^. MAVS on the mitochondrial membrane preferentially mediates the synthesis of type I IFNs, whereas the expression of type III IFNs depends on MAVS localized in peroxisomes. Accordingly, the increased abundance of peroxisomes during intestinal epithelial cell differentiation correlates with the enhanced production of IFN-λs but not IFN-β^16^. It is not entirely clear how MAVS mediates selective expression of type I versus type III IFNs depending on its subcellular localization, but the expression of type III but not type I IFNs was shown to require the activation of IRF1 in addition to IRF3^16^.

In contrast to RLRs located in the cytoplasm, TLRs sense internalized or phagocytosed viruses in endosomes or phagosomes. Unlike ubiquitously expressed RLRs, TLRs exhibit restricted expression patterns and have the highest expression in myeloid cells^15^. Among the ten human TLRs, TLR3 recognizes dsRNA, whereas TLR7 and TLR8 detect ssRNA. The endosomal localization of nucleotide-sensing TLRs, such as TLR3, TLR7, TLR8, and TLR9 (which recognizes unmethylated CpG DNA), depends on the multimembrane protein UNC93B1^17^. Ligand-stimulated TLRs recruit the cytosolic adapter molecules MYD88 and TRIF to their C-terminal Toll-interleukin receptor domains and activate both the IKKα/β/γ complex and TBK1/IKKε kinases, ultimately resulting in the induction of NFκB-dependent proinflammatory cytokines and IRF3/IRF7-dependent type I and type III IFNs.

Experiments using mouse-adapted SARS-CoV have shown that mice lacking TLR3 or the signaling adapter TRIF are more susceptible to virus-induced lung pathology and maintain higher viral titers than wild-type mice^18^. Similarly, mice deficient in TLR4 and the signaling adapter TRAM were more susceptible to mouse-adapted SARS-CoV^18^. However, unlike TLR3, which is capable of sensing viral RNA, it is not clear whether TLR4 can directly sense coronavirus components.

Thus far, evidence for the involvement of TLRs in SARS-CoV-2 infection has been provided by genetic studies. A large study involving more than 650 COVID-19 patients with severe symptoms identified mutations in *TLR3* and *UNC93B1*^19^. When infected with SARS-CoV-2 in vitro, more infection occurred in cells from patients with mutant *TLR3* alleles than in cells from healthy controls with wild-type *TLR3*. The addition of exogenous IFN-β largely removed this difference in SARS-CoV-2 infection efficiency, suggesting that IFN-β, which produced upon TLR3-mediated sensing of SARS-CoV-2, inhibits viral spread. Another genetic study that focused on young patients with severe COVID-19 identified two rare mutations in *TLR7* in four young males from two unrelated families^20^. Again, cells from the affected patients with *TLR7* mutations exhibited defective IFN-β and ISG expression after TLR7 stimulation. Notably, *TLR7* is an X-linked gene, and the loss-of-function mutation in *TLR7* or the difference in *TLR7* gene dosage between men and women may explain, at least in part, the predisposition of men to developing severe COVID-19^20,21^.

## Inhibition of IFN responses by coronaviruses

Human common-cold coronaviruses, such as HCoV-229E, induce high levels of type I IFN expression, whereas the more pathogenic and potentially lethal coronaviruses SARS-CoV, SARS-CoV-2, and MERS-CoV generally induce blunted type I IFN responses. Since the first SARS outbreak, multiple studies have demonstrated that SARS-CoV and MERS-CoV use various mechanisms to avoid type I IFN-mediated immune responses^13,22–25^. The SARS-CoV genome encodes four structural proteins (spike [S], envelope [E], membrane [M], and nucleocapsid [N]), 16 nonstructural proteins (Nsps), and other accessary proteins. Remarkably, more than one-third of SARS-CoV proteins, including structural proteins, have inhibitory effects on type I IFN-mediated antiviral immune responses^13,22–25^. The SARS-CoV-2 genome has 82% nucleotide identity with the SARS-CoV genome, and most of the SARS-CoV-2 proteins have high amino acid sequence homology with the corresponding SARS-CoV proteins, with the exception of ORF3b, ORF6, and Nsp3^14^. ORF3b is much shorter in SARS-CoV-2, with only 22 amino acids compared to 154 amino acids in SARS-CoV^14^. The ORF6 and Nsp3 proteins share 69% and 76% homology, respectively, between SARS-CoV and SARS-CoV-2^26^. Therefore, many SARS-CoV-2 proteins are expected to have inhibitory effects on type I and III IFN responses similar to those of SARS-CoV proteins. In less than a year from the start of the COVID-19 pandemic, several reports have already confirmed the antagonistic effects of SARS-CoV-2 proteins on IFN responses.

### Evasion of innate immune receptor-mediated viral sensing

SARS-CoV-2 and other coronaviruses replicate inside double membrane vesicles, preventing the recognition of dsRNA replication intermediates by cytosolic RLRs^27^. In addition, modification of the viral RNA by Nsp14 of SARS-CoV, which has guanine-N7-methyltransferase activity, mimics the 5′ cap structure of host mRNAs, allowing the efficient escape of viral RNA from detection by RIG-I^28,29^. Further modification of the cap-like structure of the viral RNA by Nsp16, which has 2′-O-methyltransferase activity, prevents MDA-5-mediated sensing of viral RNA^29^. Nsp15 is a highly conserved endonuclease in all known coronaviruses and cleaves the 5′-polyuridine sequences from negative-sense viral RNA, inhibiting the MDA-5-mediated detection of viral RNA and subsequent IFN-β production^30^.

### Inhibition of innate immune receptor signaling and IFN production

The SARS-CoV and MERS-CoV N proteins bind to the SPRY domain of the E3 ubiquitin ligase TRIM25, inhibiting TRIM25-mediated RIG-I ubiquitination and activation, thereby blocking the production of type I IFNs and increasing viral replication^31^.

The ORF9b protein of SARS-CoV has been shown to localize in mitochondria, trigger the AIP4 E3 ubiquitin ligase-mediated degradation of MAVS, and impair IFN-β production^32^. A recent study on the host protein interactomes of the individual SARS-CoV-2 proteins using HEK293T cells showed that SARS-CoV-2 ORF9b associates with mitochondrial TOM70, suggesting that ORF9b of SARS-CoV-2 may also inhibit MAVS in mitochondria^33^. The ORF9b–TOM70 interaction and ORF9b-mediated inhibition of IFN-β expression were further confirmed by ORF9b overexpression experiments^34^. In addition, a host protein interactome study showed that Nsp13 and Nsp15 interact with TBK1 and the TBK1 activator RNF41, respectively, suggesting the potential inhibition of TBK1-mediated signaling by Nsp13 and Nsp15^33^.

The Nsp3 proteins of SARS-CoV and SARS-CoV-2 have papain-like protease activity^35^. Interestingly, these proteins can also recognize ubiquitin and the ubiquitin-like modifier ISG15 and serve as deubiquitinating and deISGylating enzymes. The removal of K63-linked ubiquitin chains from TRAF3 and TRAF6 by SARS-CoV Nsp3 has been shown to inhibit TBK1 activation and IFN-β production upon TLR7 activation^35^. SARS-CoV Nsp3 has also been shown to inhibit STING-mediated production of type I IFNs by disrupting the STING–TRAF3-TBK1 interaction and IRF3 phosphorylation^36–38^. SARS-CoV and SARS-CoV-2 Nsp3 proteins share 83% sequence identity but exhibit different host substrate preferences; SARS-CoV Nsp3 predominantly targets ubiquitin chains, whereas SARS-CoV-2 Nsp3 preferentially cleaves ISG15^39^. The crystal structure of SARS-CoV-2 Nsp3 in complex with ISG15 revealed distinctive interactions between Nsp3 and the amino-terminal ubiquitin-like domain of ISG15, highlighting the high affinity and specificity of these interactions. Furthermore, upon viral infection, SARS-CoV-2 Nsp3 contributes to the cleavage of ISG15 from IRF3 and attenuates type I IFN responses^39^. Notably, inhibiting the catalytic activity of SARS-CoV-2 Nsp3 with the specific small chemical inhibitor GRL-0617 improved the antiviral IFN responses and reduced viral replication in infected cells^39^.

SARS-CoV-2 M protein physically associates with RIG-I, TRAF3, TBK1, and IKKε and sequesters these proteins in membrane-associated compartments, preventing the TBK1/IKKε-dependent activation of IRF3/IRF7 and production of type I IFNs^40^.

Overexpression of SARS-CoV Nsp1 inhibits the virus-induced dimerization of IRF3 and IFN-β promoter activation^41^. In addition, SARS-CoV and SARS-CoV-2 Nsp1 proteins interact with the 40S ribosomal subunit and almost completely shutdown host protein translation, including that of IFN-β, suggesting that Nsp1 may inhibit IFN responses by more than one mechanism^42^. Cryo-electron microscopy analysis has demonstrated that the C-terminal helices of SARS-CoV-2 Nsp1 bind and block the mRNA entry tunnel of ribosomes^43^. Furthermore, SARS-CoV-2 Nsp1 efficiently prevents the virus-induced expression of type I and type III IFNs, as well as IFN-β-stimulated ISG expression^43^. Notably, mutant Nsp1 protein lacking ribosome binding activity fails to inhibit virus-induced IFN expression, suggesting that inhibiting host protein synthesis is the major mechanism underlying the Nsp1-mediated evasion of IFN responses^43^. Although the translation of host mRNAs is globally suppressed by Nsp1, the 5′ leader sequence in viral mRNAs appears to spare these transcripts from Nsp1-mediated translational inhibition, allowing the preferential production of viral proteins^44^.

Overexpression of SARS-CoV ORF3b has been shown to inhibit IFN-β expression in Sendai virus-infected cells, and SARS-CoV-2 ORF3 was recently shown to inhibit the nuclear translocation of IRF3^45,46^. Despite the truncation of ORF3b in SARS-CoV-2 compared to its ortholog in SARS-CoV, SARS-CoV-2 ORF3b has a stronger inhibitory effect on the virus-induced nuclear translocation of IRF3 and resulting IFN-β production^46^. The original SARS-CoV ORF3b has a C-terminal nuclear localization sequence (NLS) and is equally distributed between the cytosol and nucleus, whereas SARS-CoV-2 ORF3b lacks the NLS and remains in the cytosol, presumably sequestering IRF3 outside the nucleus more efficiently than SARS-CoV ORF3b. By screening ~17,000 SARS-CoV-2 sequences, the authors of the study isolated a longer variant (56 amino acids versus 22) of SARS-CoV-2 ORF3b in two related and critically ill patients and showed that this variant has an even more enhanced inhibitory effect on IFN-β production than the original SARS-CoV-2 ORF3b. Because the longer variant also harbors a missense mutation (L24M), the mechanism underlying the increased inhibitory effect is not yet clear^46^.

ORF6 of SARS-CoV-2 also interferes with the nuclear translocation of IRF3^47^. SARS-CoV-2 ORF6 inhibits IFN-β promoter activity, even after the overexpression of a constitutively active form of IRF3, confirming that it prevents IFN-β production downstream of IRF3 activation^47^.

The NS4b protein (encoded by *ORF4b*) of MERS-CoV is a phosphodiesterase superfamily protein that primarily localizes in the nucleus. Mutation in the catalytic site or NLS of NS4b leads to the increased production of virus-stimulated IFN-β and IFN-λ1, as well as ISGs, demonstrating that NS4b normally inhibits type I and III IFN expression^48^.

### Inhibition of IFN receptor signaling and ISG expression

SARS-CoV ORF3a overexpression has been reported to induce the phosphorylation, ubiquitination, and lysosome-mediated degradation of IFNAR1 in Huh7 cells^49^. However, the effect of decreased IFNAR1 expression on type I IFN signaling and ISG induction was not directly measured in the same study.

The Nsp1, ORF3b, and ORF6 proteins of SARS-CoV not only inhibit IFN-β expression in virus-infected cells but have also been shown to prevent ISRE activation and ISG expression in the presence of exogenous recombinant IFNs, suggesting that these factors can block IFNAR signaling^41,45^. Specifically, SARS-CoV Nsp1 inhibits the tyrosine phosphorylation of STAT1 and subsequent activation of ISRE promoter activity^41^. In contrast, neither ORF3b nor ORF6 of SARS-CoV affects the IFN-β-induced tyrosine phosphorylation of STAT1^45^. However, SARS-CoV ORF6 but not ORF3b inhibits the nuclear translocation of STAT1^45^. ORF6 has also been shown to localize in the endoplasmic reticulum and Golgi membrane of SARS-CoV-infected cells and disrupt nuclear import complex formation by tethering karyopherin α2 and karyopherin β1 to the membrane^50^. Similar to SARS-CoV ORF6, SARS-CoV-2 ORF6 inhibits the IFN-β-stimulated nuclear translocation of STAT1 without affecting STAT1 phosphorylation, resulting in the inhibition of ISRE promoter activity and ISG expression^47^.

Overexpression of the SARS-CoV-2 N protein has been shown to inhibit the phosphorylation and nuclear translocation of STAT1 and STAT2 in HEK293T cells infected with Sendai virus or stimulated with recombinant IFN-β, suggesting that the N protein inhibits IFNAR signaling^51^. Furthermore, in Huh7 cells overexpressing the N protein, the replication of SARS-CoV-2 RNA was enhanced, and ISG expression was significantly attenuated^51^.

Nsp1, Nsp7, Nsp9, and Nsp16 globally affect host protein synthesis and transport, thereby preventing efficient ISG expression^43,44^. As mentioned previously, Nsp1 blocks mRNA entry into the ribosome and consequently inhibits host protein translation^43^. Nsp16 binds to pro-mRNA recognition sites in the U1 and U2 components of the spliceosome and disrupts global mRNA splicing, including that of mRNAs for multiple IFN-responsive genes^44^. SARS-CoV-2 NSP7 and NSP9 inhibit secretory and membrane protein trafficking by binding to the signal recognition particle (SRP)^44^. The SRP normally interacts with the signal peptides present in nascent proteins destined for secretion or membrane integration and mediates their translocation into the endoplasmic reticulum. The binding of NSP7 and NSP9 to the SRP disrupts the SRP-dependent secretion of host proteins, diminishing IFN responses upon SARS-CoV-2 infection^44^.

### Suppression of ISG functions

Oligoadenylate synthetase (OAS) is one of the major ISGs with antiviral functions^52^. OAS synthesizes 2′,5′-oligoadenylate from ATP upon the binding of viral dsRNA, and the resulting 2′,5′-oligoadenylates trigger the homodimerization and activation of ribonuclease L (RNase L), which is constitutively expressed in an inactive form in the steady state. Activated RNase L cleaves host and viral ssRNAs, leading to translational arrest and subsequent cell death and preventing viral replication and spread. MERS-CoV NS4b not only inhibits the virus-induced expression of type I and III IFNs but also cleaves 2′,5′-oligoadenylates via its phosphodiesterase activity, thereby antagonizing the antiviral effects of the OAS–RNase L pathway^53^.

The diverse inhibitory functions exhibited by the large number of SARS-CoV and SARS-CoV-2 proteins at every level of host IFN responses, ranging from initial viral sensing to the antiviral effector functions of ISGs (Table 1), suggest that it is highly beneficial for these viruses to thwart host IFN pathways. However, these inhibitory effects only work within virus-infected cells in which viral proteins are present in the cytoplasm, not in noninfected innate immune cells, such as monocytes and DCs.

**Table 1:** Coronavirus proteins that inhibit virus-induced interferon responses.

Mode of action	Protein	Virus	Function	Evasion mechanisms	Reference
Evasion of sensing by host innate immune receptors	Nsp14	SARS-CoV	Guanine-N7-methyltransferase	Modification of viral RNA and prevention of RIG-I-mediated viral sensing	^28,29^
	Nsp15	SARS-CoV	Endonuclease	Cleavage of viral RNA and prevention of viral sensing	^30^
	Nsp16	SARS-CoV	2′-O-methyl-transferase	Modification of viral RNA and prevention of MDA-5-mediated viral sensing	^29^
Inhibition of innate immune receptor signaling and IFN production	Nsp1	SARS-CoV, SARS-CoV-2	Binding to small ribosomal subunit	Shutdown of host mRNA translation, including mRNAs for IFNs and ISGs	^42–44^
			?	Inhibition of IRF3 dimerization (SARS-CoV)	^41^
	Nsp3	SARS-CoV, SARS-CoV-2	Papain-like protease; deubiquitinating and deISGylating activity	Removal of K63-linked ubiquitin chains from TRAF3 and TRAF6 (SARS-CoV) and detachment of ISG15 from IRF3 (SARS-CoV-2)	^35,39^
	N	SARS-CoV, MERS-CoV	Binding to TRIM25	Inhibition of TRIM25-mediated RIG-I ubiquitination and activation	^31^
	M	SARS-CoV	Association with RIG-I, TRAF3, TBK1, and IKKε	Prevention of the TBK1/IKKε-dependent activation of IRF3/IRF7	^40^
	ORF3b	SARS-CoV, SARS-CoV-2	?	Inhibition of IRF3 nuclear translocation	^45,46^
	OFR6	SARS-CoV, SARS-CoV-2	Binding to karyopherin α2 and karyopherin β1 (?)	Inhibition of IRF3 nuclear translocation	^47^
	ORF9b	SARS-CoV, SARS-CoV-2	Association with MAVS and TOM70 in mitochondria	AIP4-mediated degradation of MAVS, TRAF3, and TRAF6 (SARS-CoV)	^32,34^
	NS4b	MERS-CoV	Phosphodiesterase	Inhibition of type I and III IFN production	^48^
Inhibition of IFNAR signaling and ISG expression	Nsp1	SARS-CoV, SARS-CoV-2	?	Inhibition of STAT1 phosphorylation (SARS-CoV)	^41^
			Binding to small ribosomal subunit	Shutdown of host mRNA translation, including mRNAs for IFNs and ISGs	^42–44^
	Nsp7	SARS-CoV-2	Binding to SRP	Inhibition of host protein secretion, including that of IFNs and ISGs	^43,44^
	Nsp9	SARS-CoV-2	Binding to SRP	Inhibition of host protein secretion, including that of IFNs and ISGs	^43,44^
	Nsp16	SARS-CoV-2	Binding to spliceosome components	Disruption of pre-mRNA splicing and the resulting inhibition of host protein expression, including that of ISGs	^43,44^
	ORF3a	SARS-CoV	?	Degradation of IFNAR1	^49^
	ORF3b	SARS-CoV	?	Inhibition of ISRE activation and ISG expression	^45^
	ORF6	SARS-CoV, SARS-CoV-2	Binding to karyopherin α2 and karyopherin β1	Disruption of the nuclear import complex and inhibition of STAT1 nuclear translocation	^45,47,50^
	N	SARS-CoV-2	?	Inhibition of STAT1/STAT2 phosphorylation	^51^
Suppression of ISG function	NS4b	MERS-CoV	Phosphodiesterase	Degradation of 2′,5′-polyandenylates and inhibition of OAS/RNase L activity	^53^

## Dysregulated IFN responses and hyperinflammation in COVID-19 patients

Type I IFNs have been reported to play critical roles in the defense against SARS-CoV-2 infection and the prevention of severe COVID-19. In a genetic study of COVID-19 patients, mutations in genes in the type I IFN pathway were shown to be enriched in patients with life-threatening symptoms compared to control patients with asymptomatic or mild SARS-CoV-2 infection. Specifically, 23 of 659 (3.5%) patients with severe symptoms harbored deleterious genetic variants of *TLR3*, *UNC93B1*, *TICAM1* (encoding TRIF), *TBK1*, *IFR3*, *IFR7*, *IFNAR1*, and *IFNAR2*^19^. These mutations were experimentally proven to cause the loss of expression or a loss of function, and as expected, no or extremely low levels of type I IFNs were detected in the blood of patients harboring these mutations^19^.

The importance of type I IFN-mediated immunity in COVID-19 was also shown in a study that measured autoantibodies against type I IFNs in COVID-19 patients. Strikingly, 101 of 987 (10.2%) patients with life-threatening symptoms had autoantibodies against IFN-α, IFN-ω, or both. In contrast, these autoantibodies were present in none of the 663 patients with asymptomatic or mild SARS-CoV-2 infection and in only 4 of 1227 (0.33%) individuals in the general population^54^. These autoantibodies were able to neutralize high concentrations of type I IFNs, thwarting the ability of IFNs to prevent SARS-CoV-2 infection in vitro. Moreover, autoantibodies were already present in blood samples acquired from some patients before SARS-CoV-2 infection, and very low levels of blood IFN-α, if any, were generally detected during the acute phase of infection in patients with autoantibodies. Interestingly, none of the patients with autoantibodies against type I IFNs or mutations in genes in the type I IFN pathway seem to have a history of life-threatening viral infection before COVID-19. This finding suggests that type I IFNs may play more important roles in protecting hosts against SARS-CoV-2 infection than other viral infections^19,54^.

Transcriptomic studies of COVID-19 patients also show that IFN responses are impaired in patients with severe disease. An early study reported that type I IFN responses were limited in postmortem lung tissues from lethal cases of COVID-19, which was consistent with the data from bronchial epithelial cells infected in vitro and ferrets infected in vivo with SARS-CoV-2^55^. Another study also reported that type I IFN responses were highly impaired in the peripheral blood of patients with severe or critical COVID-19, as indicated by low levels of type I IFNs and ISGs, despite increased levels of TNF-, IL-6-, and NFκB-driven inflammatory responses^56^.

Paradoxically, robust type I IFN responses have also been reported in patients with severe COVID-19^57^. In a single-cell RNA sequencing (scRNA-seq) study of peripheral blood mononuclear cells (PBMCs) from COVID-19 patients, strong upregulation of numerous ISGs was observed in a patient with severe disease compared to patients with mild disease^58^. Moreover, a transcriptome study of bronchoalveolar lavage fluid from COVID-19 patients showed increased expression of numerous ISGs in addition to marked increases in proinflammatory and chemokine genes, suggesting proinflammatory roles of upregulated ISGs in COVID-19^59^. In another scRNA-seq study of PBMCs from COVID-19 patients, various ISGs were upregulated, especially in classical monocytes^60^. A longitudinal analysis demonstrated that IFN-α in peripheral blood was sustained at high levels in patients with severe COVID-19, whereas IFN-α levels declined in patients with moderate COVID-19 during the course of the infection^61,62^.

We also previously identified the role of type I IFN responses in the development of severe COVID-19^63^. ScRNA-seq analysis was performed using PBMCs from patients with mild or severe COVID-19 or severe influenza. COVID-19 patients had unique hyperinflammatory signatures across all types of peripheral blood immune cells, particularly increases in TNF- and IL-1β-driven inflammatory responses, whereas IFN responses were dominant in severe influenza patients. Interestingly, type I IFN responses coexisted with TNF- and IL-1β-driven inflammatory responses in classical monocytes from severe COVID-19 patients but not in those from mild COVID-19 patients, indicating that type I IFNs contribute to exacerbation of TNF- and IL-1β-driven inflammation during the development of severe COVID-19. Notably, severe COVID-19-specific signatures, including various ISGs discovered in that study, were also significantly enriched in the transcriptome of postmortem lung tissue from COVID-19 patients^55^, confirming that type I IFN responses are upregulated in severe COVID-19 cases.

Recently, dysregulated type I and III IFN responses were identified in patients with COVID-19^64^; the production of type I and III IFNs was diminished and delayed in COVID-19 patients, whereas proinflammatory cytokines, including TNF, IL-6, and IL-8, were produced before type I and III IFNs and for a prolonged period. Importantly, prominent IFN signatures were observed in critically ill patients in addition to hyperinflammation. In contrast, type I and III IFN responses were strongly induced at an early time point in patients with influenza.

Paradoxical proinflammatory roles of type I IFNs were previously described in a murine model of SARS^65^. In SARS-CoV-infected BALB/c mice, a delayed but considerable type I IFN response induced the accumulation of monocytes–macrophages and the production of proinflammatory cytokines, leading to lethal pneumonia, vascular leakage, and insufficient T-cell responses. Proinflammatory effects of type I IFNs have also been shown in SARS-CoV-2-infected mice with adeno-associated virus-mediated expression of human ACE2^66^. Using IFNAR^−/−^ and IRF3/7^−/−^ mice, the study showed that type I IFN responses were required for the recruitment of proinflammatory monocytes–macrophages into SARS-CoV-2-infected lungs.

Type I IFNs can exacerbate the inflammatory response via epigenetic molecular mechanisms. TNF has a tolerizing effect on monocytes, desensitizing these cells to additional TLR stimulation as a regulatory mechanism that limits excessive inflammation^67^. Intriguingly, type I IFNs nullify the tolerizing effect of TNF and render monocytes responsive to additional TLR signals via epigenetic mechanisms^68^. This epigenetic regulation involves the upregulation of a gene module that is unresponsive to TLR stimulation due to TNF-induced tolerance but becomes responsive to TLR signals in the presence of type I IFNs; however, this gene module is not directly upregulated by type I IFNs. Importantly, this gene module was significantly enriched in the transcriptome of classical monocytes from patients with severe COVID-19, indicating that the type I IFN response can enhance TLR-mediated inflammation by disrupting the TNF-induced tolerance to TLR signals in monocytes from patients with severe COVID-19^63^.

**Fig. 3:**
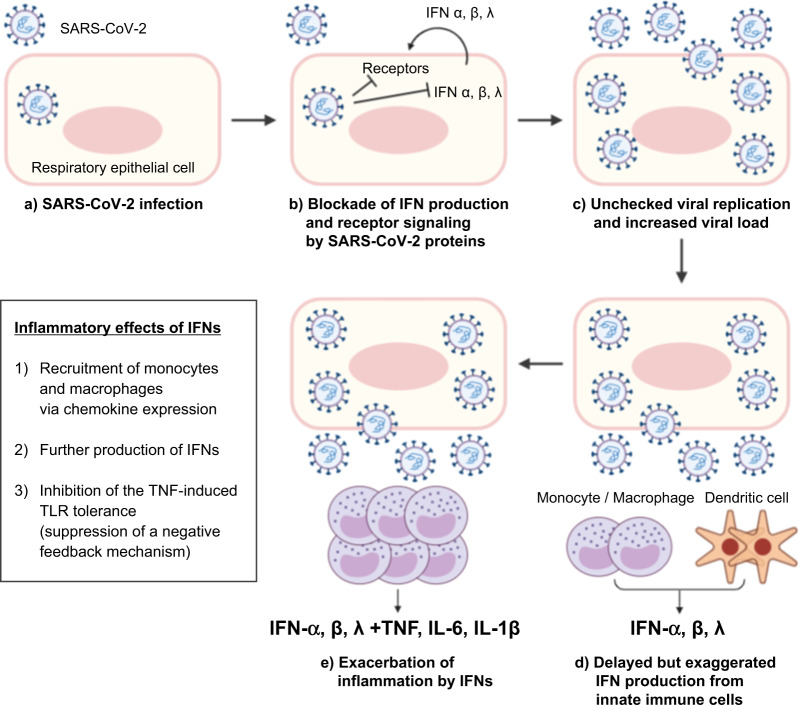
Hypothesis of how delayed but exaggerated type I IFN responses are involved in hyperinflammation and contribute to the severe progression of COVID-19. After respiratory epithelial cells are infected (**a**), SARS-CoV-2 proteins block type I and III interferon (IFN) responses (**b**). The viral load increases (**c**) and uninfected innate immune cells, such as monocytes, macrophages, and dendritic cells, are stimulated by viral components via Toll-like receptors and produce type I and III IFNs (**d**). Type I and III IFNs further induce the accumulation and activation of monocytes and macrophages, leading to the production of large amounts of IFNs and proinflammatory cytokines (**e**). Type I IFNs also enhance TNF-mediated inflammation by disrupting TNF-induced tolerance to TLR stimulation in monocytes and macrophages.

We propose a hypothesis that explains the contradictory roles of type I IFN responses reported in patients with severe COVID-19 (Fig. 3). After infecting respiratory epithelial cells, SARS-CoV-2 begins to produce its proteins and replicates. Viral proteins block type I and III IFN responses by inhibiting innate recognition of the virus, the production of IFNs, and the IFN signaling pathway. Consequently, the viral load increases due to the rapid replication of SARS-CoV-2 without efficient control of viral spread by type I and III IFN responses. Uninfected immune cells, such as monocytes, macrophages, and DCs, are stimulated by viral components via TLRs and produce large amounts of type I IFNs. The production of type I IFNs from these immune cells is not counteracted by SARS-CoV-2 proteins because these immune cells do not have viral proteins in their cytoplasm. Large amounts of type I IFNs further induce the accumulation and activation of monocytes and macrophages, leading to the production of proinflammatory cytokines. At the same time, type I IFNs enhance TNF-mediated inflammation by disrupting TNF-induced tolerance of monocytes and macrophages to TLR stimulation. This hypothesis explains how delayed but exaggerated type I IFN responses are involved in hyperinflammation and contribute to the severe progression of COVID-19. However, type I IFN responses might vary among individuals due to genetic and immunological factors^19,20,54^.

## Perspectives

Because of the broad antiviral activities of type I IFNs, recombinant IFN-α and IFN-β are being clinically investigated for the treatment of COVID-19 patients. Recently, the safety and efficacy of inhaled nebulized IFN-β1a was reported in COVID-19 patients^69^. Moreover, pegylated IFN-λs are also being clinically studied for the treatment of COVID-19 patients because IFN-λs are expected to exert antiviral effects without inflammatory effects. However, given that hyperinflammation may be exacerbated by IFN responses, the use of type I or III IFNs for the treatment of COVID-19 patients needs to be evaluated cautiously in clinical studies, particularly for patients in the late stages of COVID-19. In addition, type I or III IFN-associated biomarkers that predict the prognosis of patients with COVID-19 need to be developed to improve the management of patients. In conclusion, further investigation and a more precise understanding of the exact roles of type I and III IFNs in SARS-CoV-2 infection are needed to facilitate the effective treatment of patients with COVID-19.
